# Supplementary figures and images for: Simulating the effect of evaluation unit size on eligibility to stop mass drug administration for lymphatic filariasis in Haiti
Source: PLoS Negl Trop Dis. 2022 Jan 28;16(1):e0010150. doi: 10.1371/journal.pntd.0010150 (PMC8827424; doi:10.1371/journal.pntd.0010150)

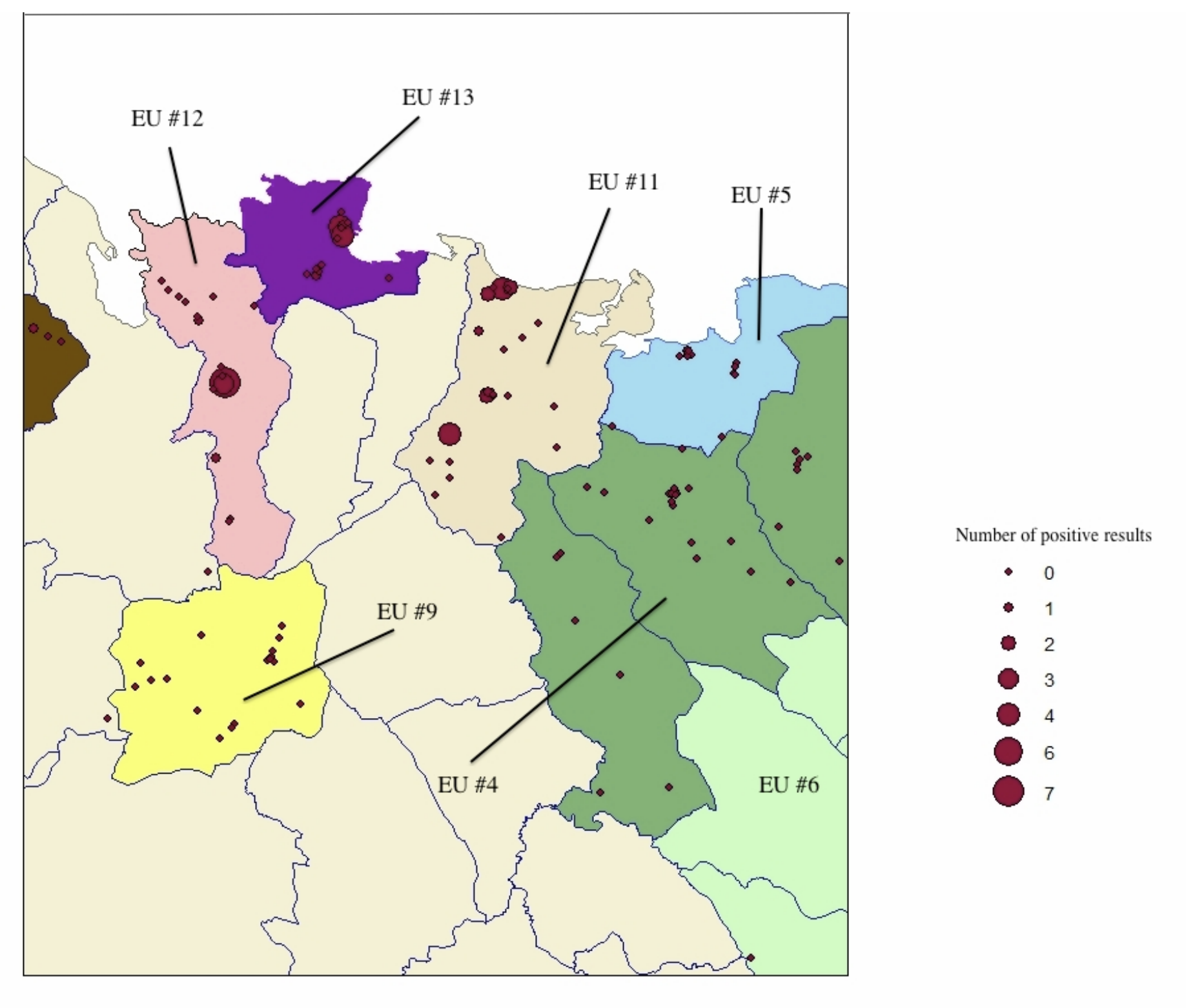

Supplement: S1 Fig — The administrative division shapefile that served as a base map is available at https://data.humdata.org/dataset/777e8b06-337f-4295-80bc-ca1515244215/resource/9b57a285-e12f-4d1a-b167-676d96a2b4af/download/hti_adm_cnigs_20181129.zip; the shapefile with Evaluation Unit number as an attribute is available for download https://doi.org/10.15139/S3/JUUSHC. Assessment Survey data. (TIF) [file pntd.0010150.s001.tif]
