# Supplementary material for: Simulating the effect of evaluation unit size on eligibility to stop mass drug administration for lymphatic filariasis in Haiti
Source: PLoS Negl Trop Dis. 2022 Jan 28;16(1):e0010150. doi: 10.1371/journal.pntd.0010150 (PMC8827424; doi:10.1371/journal.pntd.0010150)
Supplement: S1 Table — Table adapted from [13]. (PDF) [file pntd.0010150.s002.pdf]

Population Surveyed (N)	Systematic sample (for districts with <40 schools)				Cluster sample (for districts with ≥40 schools)	
	Critical Cutoff	Sample Size	Range of α	Power	Critical Cutoff	Sample Size
≥2,000	2	320	3.3%-4.5%	35.7%-37.9%	3	480
1,000-1,999	2	300	3.4%-5.3%	38.2%-44%	3	450
750-999	1	220	3.8%-5.5%	34.3%-37.6%	NA	NA
500-749	1	210	3.4%-6.3%	30.2%-37.1%	NA	NA
<500	0.02*N	Census (N)			NA	NA
