# Supplementary material for: Simulating the effect of evaluation unit size on eligibility to stop mass drug administration for lymphatic filariasis in Haiti
Source: PLoS Negl Trop Dis. 2022 Jan 28;16(1):e0010150. doi: 10.1371/journal.pntd.0010150 (PMC8827424; doi:10.1371/journal.pntd.0010150)
Supplement: S2 Table — (PDF) [file pntd.0010150.s003.pdf]

Table S2. Comparison of observed 2015 Haiti Transmission Assessment Survey results in 13 Evaluation Units and simulated results using bootstrapping

Evaluation Unit #	# Children tested in Transmission Assessment Survey	# Positive results	Critical cutoff	Observed Transmission Assessment Survey decision	Expected true prevalence (upper 1-sided Confidence interval)	Expected transmission assessment conclusion	% of replicates passing Transmission Assessment Survey simulation (out of 1,000)
2	1659	3	18	Pass	0.18% (0.36%)	Pass	100%
3	1231	2	14	Pass	0.16% (0.32%)	Pass	100%
4	1528	0	18	Pass	0 (0)	Pass	100%
5	364	1	3	Pass	0.26% (0.78%)	Pass	69.4%
6	1617	2	18	Pass	0.13% (0.25%)	Pass	100%
7	551	0	6	Pass	0 (0)	Pass	100%
8	1587	2	18	Pass	0.13% (0.25%)	Pass	100%
9	587	0	6	Pass	0 (0)	Pass	100%
10	672	0	7	Pass	0 (0)	Pass	100%
11	858	19	9	Fail	2.17% (3.31%)	Fail	4.4%
12	1037	15	11	Fail	1.37% (2.29%)	Fail	29.9%
13	1984	19	20	Pass	0.95% (1.29%)	Pass	95.3%
14	1414	10	16	Pass	0.71% (1.01%)	Pass	99.1%

Number of children tested in Transmission Assessment Survey is the number of positive and negative Immunochromatographic card test results that were recorded in the Evaluation Unit during the Transmission Assessment Survey. If the number of positive results in the Evaluation Unit is greater than the critical cutoff, the observed Transmission Assessment Survey decision is to fail; else, it is to pass. The expected true prevalence is the weighted average of prevalence in the EUs comprising the combo-EU. The expected Transmission Assessment Survey decision is to fail the combo-EU if the upper one-sided 95% confidence interval of the expected true prevalence is greater than or equals 2%, and to pass otherwise.
