# Supplementary material for: Simulating the effect of evaluation unit size on eligibility to stop mass drug administration for lymphatic filariasis in Haiti
Source: PLoS Negl Trop Dis. 2022 Jan 28;16(1):e0010150. doi: 10.1371/journal.pntd.0010150 (PMC8827424; doi:10.1371/journal.pntd.0010150)
Supplement: S3 Table — (PDF) [file pntd.0010150.s004.pdf]

S3 Table. Distribution of positive Immunochromatographic card test results within Evaluation Units.

Evaluation Unit #	# Children Tested	# Positive Results	# Schools with 0 positive ICT	# Schools with 1 positive ICT	# Schools with 2 positive ICT	# Schools with 3 positive ICT	# Schools with 4 positive ICT	# Schools with 5 positive ICT	# Schools with 6 positive ICT	# Schools with 7 positive ICT
1	1494	0	19	0	0	0	0	0	0	0
2	1659	3	44	1	1	0	0	0	0	0
3	1231	2	51	2	0	0	0	0	0	0
4	1528	0	45	0	0	0	0	0	0	0
5	364	1	15	1	0	0	0	0	0	0
6	1617	2	240	2	0	0	0	0	0	0
7	551	0	25	0	0	0	0	0	0	0
8	1587	2	45	2	0	0	0	0	0	0
9	587	0	24	0	0	0	0	0	0	0
10	672	0	30	0	0	0	0	0	0	0
11	858	19	22	3	4	0	2	0	0	0
12	1037	15	30	5	0	1	0	0	0	1
13	1984	19	25	3	1	0	2	0	1	0
14	1414	10	25	6	2	0	0	0	0	0
