# Supplementary material for: Simulating the effect of evaluation unit size on eligibility to stop mass drug administration for lymphatic filariasis in Haiti
Source: PLoS Negl Trop Dis. 2022 Jan 28;16(1):e0010150. doi: 10.1371/journal.pntd.0010150 (PMC8827424; doi:10.1371/journal.pntd.0010150)
Supplement: Attachment — Submitted filename: Response to reviewers_PLOS NTDS.docx [file pntd.0010150.s005.docx]

Katherine Gass, PhD, MPH

Neglected Tropical Disease Support Center

The Task Force for Global Health

330 West Ponce de Leon

Decatur, GA 30030

October 29, 2021

Dear Dr. Coffeng and Reviewers,

Thank you for taking the time to review our manuscript entitled, "Simulating the effect of evaluation unit size on eligibility to stop mass drug administration for lymphatic filariasis in Haiti" and considering it for publication in PLOS Neglected Tropical Diseases. We feel that all three reviewers have provided valuable insights that have served to strengthen the manuscript. We have made an attempt to incorporate all the feedback from the reviewers in the revised version of the manuscript that we now submit.

Please find specific responses to each of the reviewer’s comments below.

Sincerely,

Katherine Gass

**Specific Responses to Reviewers**

With the revised submission, please include the original data in a form that allows the results to be checked and replicated. The reference to an aggregate summary of the data provided in reference 12 is not sufficient. If there are concerns to make the original microdata available to readers, if possible, please aggregate the microdata to a level that allows the bootstrap analysis to be reproduced, e.g., in the form of the number of types of test result per school (positives, negatives, indeterminate). This would allow the authors to at least drop the age and sex-related data, which based on the provided information, did not seem to play a role in the bootstrap procedure. However, if age and sex did play a role in the bootstrap procedure, the microdata should be made available without aggregation.

Response: Thank you for this recommendation. We made available aggregate data by Evaluation Unit, school, and test result.

I further encourage the authors to reconsider dropping EU #1 from the bootstrap simulations ("because an error in the original dataset made it impossible to recreate the school-level results"). Although the nature of the error is not fully clear, it sounds like a technical problem. Given that bootstrapping is a well-established technique, it should be possible to resolve a technical issue and/or find help to resolve it. Or, if the authors can provide more details about the problem in their response, perhaps the reviewers can provide a suggestion.

Response: Thank you for this comment. We apologize for not making the problem clearer. At the time that the dataset was put together, there was an error in coding school numbers in EU #1. Schools 1 through 16 were all coded as “1,” making it impossible to disaggregate the results by school. We do not see a work around, which led to decision to exclude EU #1.

We added this elaboration in lines 242 - 244 of the manuscript.

Reviewer's Responses to Questions

<b>Key Review Criteria Required for Acceptance?</b></br></br>
As you describe the new analyses required for acceptance, please consider the following:</br></br>

<b>Methods</b></br></br>
-Are the objectives of the study clearly articulated with a clear testable hypothesis stated?</br>
-Is the study design appropriate to address the stated objectives?</br>
-Is the population clearly described and appropriate for the hypothesis being tested?</br>
-Is the sample size sufficient to ensure adequate power to address the hypothesis being tested?</br>
-Were correct statistical analysis used to support conclusions?</br>
-Are there concerns about ethical or regulatory requirements being met?</br></br>

Reviewer #1: Methods were clearly defined and appropriate.

Reviewer #2: (No Response)

Reviewer #3: The objectives of the study are clearly defined. The authors test whether reasonable combinations of evaluation units would yield different programmatic decisions. The data and methods are appropriate for the questions, and the decisions to exclude some of the observed data from the deeper analysis are reasonable. One recommendation would be to add clarity on how additional clusters or children from clusters were drawn for the bootstrapping analysis. It seems odd that an analysis using totally sufficient samples could result in too small of a sample using essentially the same design.

Thank you for pointing out the lack of clarity in the presentation of this method. The insufficient sample size may result if schools selected through bootstrapping have, by chance, a smaller than average number of children that were tested – this is due to sampling variability. We’ve included an example of one such replicate in the boxplot below (note that the mean sample size per school is smaller than what was seen in the full observed data). In this replicate, we would have to bootstrap additional schools until we reached the desired sample size.

Please note that the way bootstrapping was performed here is consistent with the way that TAS is done; sometimes additional schools have to be sampled if the desired sample size is not reached when a disproportionate number of small schools are randomly selected. We added some of these details in sub-section “Bootstrapping” (lines 227 - 230). We hope that this is now sufficiently clear.

--------------------

<b>Results</b></br></br>
-Does the analysis presented match the analysis plan?</br>
-Are the results clearly and completely presented?</br>
-Are the figures (Tables, Images) of sufficient quality for clarity?</br></br>

Reviewer #1: General comment re: presentation of results:
I know “Desired programmatic decision” is defined, however it seems odd to state that any desired programmatic decision is to “Fail” – and to see that written in Table 3 just seems odd. If other language could be used to describe the same phenomena, I recommend using different language here (and in the text) so the layperson doesn’t actually ever think a program would desire to fail. Perhaps just drop the word “Desired”? Or replace it with "Purported"?

Response: Thank you for this observation. We completely agree – we would certainly not desire to “Fail” an EU! We dropped the word “Desired” throughout the manuscript, and just referred to it as “Programmatic decision.”

Reviewer #2: (No Response)

Reviewer #3: The tables and figures are clear and informative. The results are well described.
Response: Thank you very much!
--------------------

<b>Conclusions</b></br></br>
-Are the conclusions supported by the data presented?</br>
-Are the limitations of analysis clearly described?</br>
-Do the authors discuss how these data can be helpful to advance our understanding of the topic under study?</br>
-Is public health relevance addressed?</br></br>

Reviewer #1: Conclusions and considerations for programmatic decisions are clearly stated.

Reviewer #2: (No Response)

Reviewer #3: The conclusions suit the results and the scope of the analysis. The authors clearly describe the limitations of their work and the underlying data. The relevance and applicability are well treated.

--------------------

<b>Editorial and Data Presentation Modifications?</b></br><br/>
Use  this section for editorial suggestions as well as relatively minor modifications of existing data that would enhance clarity. If the only modifications needed are minor and/or editorial, you may wish to recommend “Minor Revision” or “Accept”.

Reviewer #1: Minor suggested revisions below:
Line 15, replace Hispaniola Program with Hispaniola Initiative Thank you, replaced.
Line 91: Recommend moving sentence “The only guidance…” before the sentence that starts on line 88 “An EU should be comprised…” Thank you. We moved the sentence as suggested to line 89, and removed the first part of the sentence for better flow.
Line 149: First use of STH – but hasn’t been spelled out previously. Thank you for catching this. We replaced STH with the full term (soil-transmitted helminthiasis), as we don’t use the abbreviation anywhere else in the paper.
Line 276: “Error” Reference source not found.” Is printed in lieue of reference. Thank you – we updated the reference.
Line 281: Missing word “of” between “number students”. Thank you, addressed.
Line 283: Recommend formatting Table 1 to emulate Tables 2 - 4 (e.g., same font size) Thank you, adjusted font size in Table 1 to match the other tables.
Line 300: Recommend adding the words “passed the TAS but” btwn “EU #13” and “came close to”. Thank you, added in (line 315 in the revised version).
Line 304: Capitalize the “F” in “figure 1” Thank you, done.
Line 306. Transmission Assessment Surveys and Evaluation Units could be capitalized – see Table 3 – or review all figure and table titles throughout and be consistent. Thank you, we capitalized Transmission Assessment Surveys and Evaluation Units in the figure caption, both for Figure 1 and Table 1 and Table 2 titles.
Line 337: Add “the” btwn “in majority”. Thank you, added.
Line 343: Everywhere else, “card test” is lowercase except here. Choose one and be consistent throughout. Thank you, corrected to lowercase.
Line 369: Everywhere else, “card test” is lowercase except here. Choose one and be consistent throughout. Thank you, corrected to lowercase.
Line 373: Consider replacing “stopping-treatment decisions” with “stopping-MDA decisions” or “stop-MDA decisions” as is used elsewhere in manuscript. Thank you. Replaced with “stop-MDA decision”.
Line 374: Replace TAS surveys with “TASs” – which has been used elsewhere in manuscript. Thank you, replaced with TASs as suggested.
Line 466: “stop MDA decisions was earlier hyphenated as “stop-MDA decisions” – review manuscript in entirety and be consistent. Thank you. Replaced with “stop-MDA decisions” here and also in line 252.
Line 533: S1 Figure, spell out acronyms. Thank you. Spelled out ICT and TAS.

Reviewer #2: (No Response)

Reviewer #3: (1) The abstract does not mention the mini-TAS analysis. If there is room, please briefly note that you explored alternative designs as a compromise. Thank you for this suggestion. We added a little bit of detail about mini-TAS in the “Methodology/ Principal Findings” and “Conclusions / Significance” section of the Abstract (lines 42 – 44, 50 – 52).
(2) While the use of the upper 95% CI to determine the TAS result based on bootstrapping is functionally equivalent to the LQAS, most readers will be more familiar with decision rules and cutoffs when it comes to TAS data. It may be helpful to reiterate this in the results, that the simulations produced a frequency of positive ICTs above the cutoff.

We agree, this is an important point to drive home for the readers. We have added some text to reiterate that the upper 1-sided 95% CI corresponds to where the critical cutoff is set in the TAS. (lines 103-109 and line 210)
(3) The authors note in line 256 that the mini-TAS uses "population proportionate to estimated size sampling, which results in a fixed sample size across all schools." Is this not the other way around, that the PPES sampling uses a fixed fraction rather than a fixed number?

Thank you. We recognize that, as written, the description of the mini-TAS sampling design may lead to confusion. The mini-TAS does indeed lead to a fixed *expected* sample size per cluster; we have tried to clarify why this is so in the text as follows (lines 269 - 274).

(4) There is a reference error in line 276. Thank you, we updated the reference.
(5) Table 1: perhaps include a column noting which EUs will be excluded from further analysis due to low number of positive children. Thank you. Added a star next to these units and a description below the table.
(6) Around line 414-418, the authors note that "Because the mini-TAS sample size is much smaller, a single team can typically complete sampling in two clusters (i.e., schools) per day, which may result in two- or three-fold savings in survey implementation costs." With school-based sampling and good mobilization, two clusters per day is plausible in a regular TAS. What are the estimated savings in labor and supplies that come from the smaller number of clusters and overall smaller sample size of the mini-TAS when compared to a regular TAS, particularly one done on a tighter schedule? The cost-effectiveness of switching to a mini-TAS approach is an important consideration that will ultimately determine whether this strategy is feasible and desirable. Unfortunately, such an analysis is outside the scope of this current study. Nonetheless, published experiences with both tools in Tanzania suggest that the mini-TAS costs $9,598 per EU (Gass et al, PLOS NTDs) while the cost of the TAS is $29,721 (Brady et al, PLOS NTDs). This significant saving is likely due to the reduction in the supply needs and duration of the survey when the mini-TAS is used. We’ve edited the manuscript to address this in lines 437 – 439.
(7) Lines 444-446: did you notice a difference in the results when drawing from discordant sample designs (cluster v. systematic)? Perhaps in the variance? Intuitively, it seems like the very different population structures would affect the results in some way. Thank you for bringing up this point – this is certainly something interesting to consider. Unfortunately, it’s hard to say given the low number of combination-EUs in each of the categories (we have 3 combination-EUs that are purely cluster, and the rest are mixed). Intuitively, we’d say there’s more difference in terms of potential sampling bias, but we don’t think the study at hand allows us to assess whether the differences observed are due to mixture of sampling designs or differences in distribution of ICT results across EUs.

(8) Lines 450-451: Would reducing the upper population threshold from 2 million to say 1 million or 500,000 make a difference? Please elaborate more on why epidemiological conditions (presumably, high baselines leading to persistent transmission after years of MDA) matter more than the other factors. Moreover, programs often only have baselines and perhaps an interim survey or two before Pre-TAS. Beyond increasing the frequency of interim monitoring or the number of sentinel and spot check sites, how should programs classify their baselines? I.e., does 5% go with 10%, does 10% go with 15%, etc. This may be an area of further work and another paper, but a rough +/- x% may be helpful.

This is an excellent point and part of an ongoing discussion in the LF community. We do not feel that it is possible to provide a precise threshold, or even an approximate one, because baseline prevalence should be considered in the context of other relevant epidemiologic factors. An additional challenge is that most baseline data are quite sparse, and not necessarily indicative of the highest starting prevalence in the implementation units. In response to the reviewer’s point, we have added a sentence on this topic in the discussion (Lines 481 - 484).

--------------------

<b>Summary and General Comments</b></br></br>
Use  this section to provide overall comments, discuss strengths/weaknesses of the study, novelty, significance, general execution and scholarship. You may also include additional comments for the author, including concerns about dual publication, research ethics, or publication ethics. If requesting major revision, please articulate the new experiments that are needed.

Reviewer #1: Very well-written with clear explanation of relevance to programmatic decision-making.

Reviewer #2: I have read the paper “Simulating the effect of evaluation unit size on eligibility to stop mass drug administration for lymphatic filariasis in Haiti” with great interest. It is a great piece of work with clear policy relevance for the global programme to eliminate lymphatic filariasis. I have some suggestions and minor comments

Thank you to our reviewers for your kind comments, as well as all of your extremely helpful feedback and suggestions!

SUGGESTIONS:

1.      I miss an assessment of the reproducibility of TAS results with current EU-size and guidelines. Could you do 1000 bootstraps of the data within each component EU to assess how often that EU would be classified as passing or failing TAS?

Thank you for this suggestion. We included this table in Supporting Information S2 Table.

2.      Table 1: could you add information about the number of positives per school (e.g. no positives in x schools, 1 positive in y schools, etc), so that readers have complete information to reproduce the analyses?

Thank you. We added a table as a standalone table in the Supporting information (S3 Table), as it is quite wide. Please note that we also made available the dataset with number of positive and negative ICT results by schools by EU, which should allow for reproducibility of results.

3.      Table 1: it is interesting to see how the number of children tested varies with population size. In the smallest EUs about half of the target population is being tested. This declines to about 5% in the largest EUs. Similarly, nearly all schools are surveyed in small EUs, while only 10% of schools is surveyed in the largest EUs. Explaining the rationale behind the survey sample builder is perhaps beyond the scope of this paper, but it might be useful for readers to be reminded about this and to understand under which circumstances this would be appropriate.
We appreciate the importance of clarifying how the TAS is designed and have added in details in lines 103 - 109.

MINOR COMMENTS

4.      Line 123: I’d suggest to delete the word “safely”, as the appropriateness of the decision often remains to be seen Thank you, change made.

5.      Lines 123-133: this text is written as if there was not any statement about homogeneity in WHO’s guidance for creating EUs. But there was a statement about this in the guidelines. Perhaps one or two sentences about why this has not always been effectively applied would be helpful in the stage. Was there a lack of guidance on how to define whether an area is sufficiently homogenous to be considered one EU?

Thank you - we have addressed this in lines (422 - 428). The challenge is being able to pick up a hotspot of transmission, and establishing how homogeneous or heterogeneous an EU is.

6.      Line 155-157: was the choice of EU-sizes in Haiti driven by information on homogeneity? Yes, the program considered baseline prevalence.

7.      Line 171-173: state explicitly that the homogeneity criterion was not considered in forming combo-EUs. Thank you; we added this in lines 179 – 181.

8.      Line 291-292: EU numbers don’t match to the numbers in table 1. Apologies, corrected from EU #2 to EU #3. Thank you!

9.      Figure 1: the red is difficult to see  (many points appear black to me, possibly because of the black border) Thank you for this comment. We updated the map to make the points easier to see.

10.     Line 353: did you intend to also mention the results for combo-EU B in this sentence (38.4%)? Replace “failing” by “passing” So sorry, we did intend to mention the results for combo-EU B. We added the results in the sentence (line 368).

11.     Lines 354-356: it is a bit confusing that passing rates are provided in table 3 and that failing rates are discussed in the text. I suggest to harmonize this. In the discussion of the results presented in table 3, it may be useful to point out that target population was relatively small in the two component EUs that failed TAS in the observed data. When these EUs are combined with considerably larger EUs with low baseline prevalence, these component EUs make up a minor part of the total population. Only when combined with even smaller EUs, we still get a signal. Thank you, we added this in discussion (line 399 - 404). We updated the table so that it lists failing rates to correspond to the framing in the text.

12.     Lines 397-399: I don’t understand the first part of this sentence. How does combining IUs into larger EUs prevent “misclassifying well-performing EUs as failing”? Even with small EUs they should pass the criteria. We agree and have modified the sentence (lines 415 - 416).

Reviewer #3: This paper is relevant to the NTD community. The methods are appropriate and straightforward. It is well written and clearly presented. Thank you very much!

--------------------

PLOS authors have the option to publish the peer review history of their article (<a href="<https://journals.plos.org/plosntds/s/editorial-and-peer-review-process#loc-peer-review-history>" target="_blank">what does this mean?</a>). If published, this will include your full peer review and any attached files.<br><br>

If you choose “no”, your identity will remain anonymous but your review may still be made public.<br><br>

<b>Do you want your identity to be public for this peer review?</b> For information about this choice, including consent withdrawal, please see our <a href="<https://www.plos.org/privacy-policy>" target="_blank">Privacy Policy</a>.

Reviewer #1: No

Reviewer #2: No

Reviewer #3: No
