# Supplementary material for: Simulating the effect of evaluation unit size on eligibility to stop mass drug administration for lymphatic filariasis in Haiti
Source: PLoS Negl Trop Dis. 2022 Jan 28;16(1):e0010150. doi: 10.1371/journal.pntd.0010150 (PMC8827424; doi:10.1371/journal.pntd.0010150)
Supplement: Attachment — Submitted filename: Response to reviewers_12.31.2021.docx [file pntd.0010150.s006.docx]

Dear Reviewers,

Thank you for taking the time to re-read this manuscript. I greatly appreciate your careful eyes, which caught some mistakes. I have since addressed these in the revision, as noted below.

Reviewer #1: MUST UPDATE: Table 3 numbers do not match the text; Table 3 was updated to present % fail, and line 368 incorrectly reports "6.8% and 38.4% of replicates failing TAS, respectively" - these %, if you do the math, are for passing TAS.  Recommend reviewing this complete sentence, and the Table 3 numbers, to decide what you want to present here - it could be that the % from Table 3 should be input into this sentence (alternatively, the word failing should be replaced with passing - however the complete sentence implies failure proportions are meant to be presented).  Also, comment that Table 3 updated to % fail while Table 4 still presents % pass - in the event the authors wish to be consistent.

Thank you for catching this! I have fixed the %s in line 368; in line 364 I replaced "nine" with "eight" (reviewers didn't raise this issue but I miscounted). I updated Table 4 to present failing instead of passing results for consistency as Reviewer had pointed out.

Reviewer #3: The revisions addressed the comments well and improved the paper.

--------------------

<b>Conclusions</b></br></br>
-Are the conclusions supported by the data presented?</br>
-Are the limitations of analysis clearly described?</br>
-Do the authors discuss how these data can be helpful to advance our understanding of the topic under study?</br>
-Is public health relevance addressed?</br></br>

Reviewer #3: Reviewers' concerns appear to have been well addressed.

--------------------

<b>Editorial and Data Presentation Modifications?</b></br><br/>
Use  this section for editorial suggestions as well as relatively minor modifications of existing data that would enhance clarity. If the only modifications needed are minor and/or editorial, you may wish to recommend “Minor Revision” or “Accept”.

Reviewer #3: Double check wording on line 198.

Thank you, I have rephrased the sentence in line 198 - 200.
